# Aboveground mechanical stimuli affect belowground plant-plant communication

**DOI:** 10.1371/journal.pone.0195646

**Published:** 2018-05-02

**Authors:** Ali Elhakeem, Dimitrije Markovic, Anders Broberg, Niels P. R. Anten, Velemir Ninkovic

**Affiliations:** 1 Department of Crop Production Ecology, Swedish University of Agricultural Sciences, Uppsala, Sweden; 2 Centre for Crop Systems Analysis, Wageningen University and Research, Wageningen, the Netherlands; 3 Faculty of Agriculture, University of Banja Luka, Banja Luka, Bosnia and Herzegovina; 4 Department of Molecular Sciences, Swedish University of Agricultural Sciences, Uppsala, Sweden; 5 Department of Ecology, Swedish University of Agricultural Sciences, Uppsala, Sweden; Helmholtz Zentrum Munchen Deutsches Forschungszentrum fur Umwelt und Gesundheit, GERMANY

## Abstract

Plants can detect the presence of their neighbours and modify their growth behaviour accordingly. But the extent to which this neighbour detection is mediated by abiotic stressors is not well known. In this study we tested the acclimation response of *Zea mays* L. seedlings through belowground interactions to the presence of their siblings exposed to brief mechano stimuli. Maize seedling simultaneously shared the growth solution of touched plants or they were transferred to the growth solution of previously touched plants. We tested the growth preferences of newly germinated seedlings toward the growth solution of touched (T_solution) or untouched plants (C_solution). The primary root of the newly germinated seedlings grew significantly less towards T_solution than to C_solution. Plants transferred to T_solution allocated more biomass to shoots and less to roots. While plants that simultaneously shared their growth solution with the touched plants produced more biomass. Results show that plant responses to neighbours can be modified by aboveground abiotic stress to those neighbours and suggest that these modifications are mediated by belowground interactions.

## Introduction

In nature, plants live together in communities composed of one or more species that communicate through a variety of often complex mechanisms [1]. To compensate for their sessile life form, plants have evolutionarily developed various mechanisms to perceive and to respond to their surroundings, a phenomenon denoted as plant behaviour [2–3]. Plant behaviour is complex and driven through a complex set of informative cues perceived from their neighbours [4]. Ecology theory predicts that these cue responses have been optimized to maximize performance [5–7]. These cues can be physical, e.g. changes in light quality [8], sound [9], and mechano-stimuli [10], or biochemical, e.g. organic compounds produced from shoots [11] or roots [12, 13] of surrounding plants.

Root exudates are among the most likely sources of cues because roots actively secrete a wide variety of organic compounds [13, 14], which may act as a cue to nearby plants in the detection of the competitive neighbours [4, 15]. Several studies suggest that these root-produced compounds can indicate the extent of kin-ship whether neighbouring plants are relatives (kin, siblings) or strangers, and that responding plants can accordingly adjust their patterns of biomass allocation [13, 16–18].

Plants are exposed to a range of mechano stimuli from their neighbours, *e*.*g*. touching caused by wind, the hyponastic movement of leaves [19], circumnutating of plant organs or phototropism. These mechano stimuli can act as cues of neighbour presence [19]. Canopy shyness in trees is a famous example of a plant response to mechano stimuli induced by neighbours. It is believed that canopies of trees stop expanding when they touch canopies of neighbouring trees [20]. Recent studies have shown that modest touching of leaves can cause changes in the biomass allocation strategy and alter the chemical composition of the emitted compounds [21, 22]. Still, it is unknown whether and how aboveground plant-plant communication through mechano-stimuli (*e*.*g*. leaf touching) may have implications on belowground interactions in a detection of neighbours.

**Fig 1:**
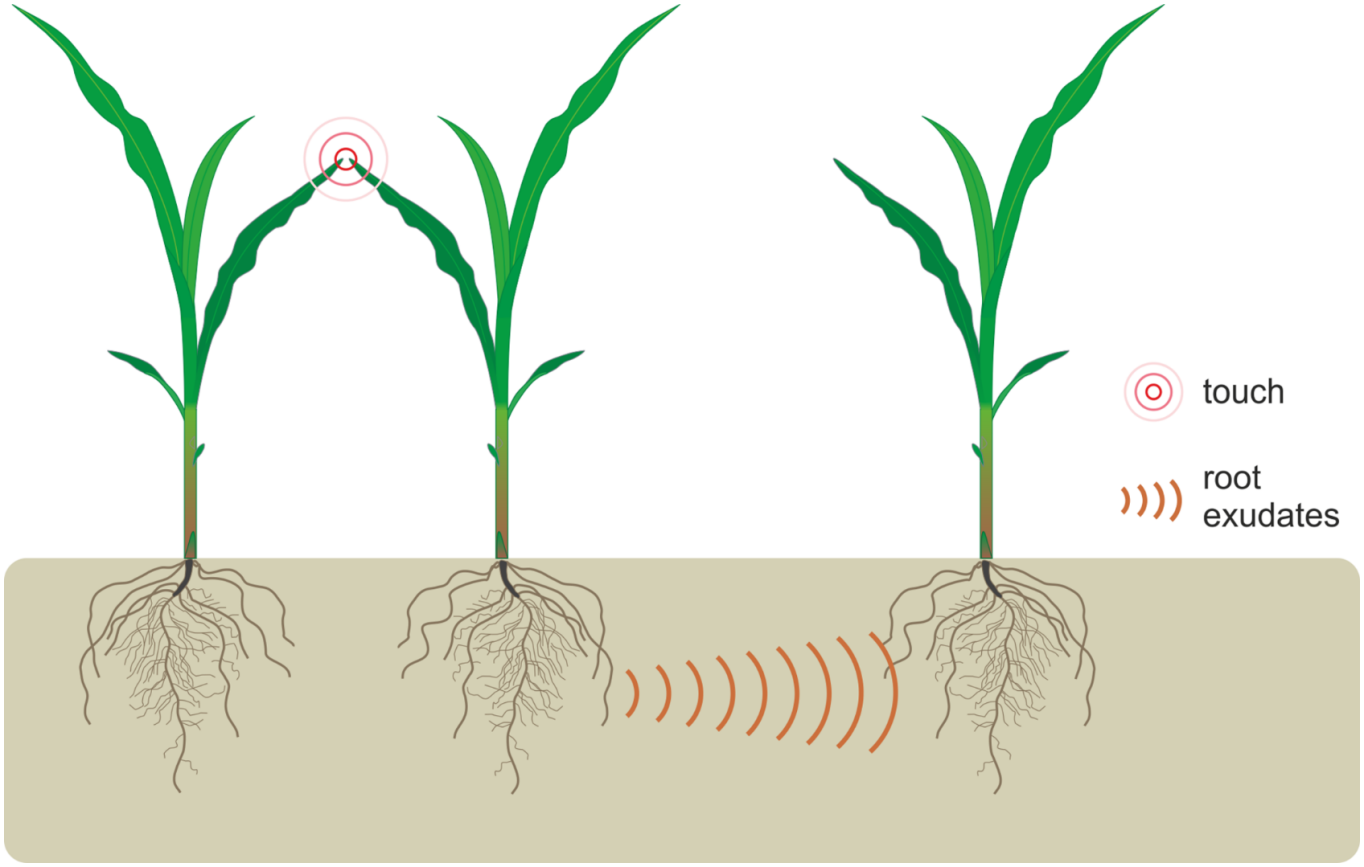
Graphical illustration of above ground interactions between neighboring plants by light touch and their effect on below-ground communication.

The aim of this study was to test whether aboveground plant-plant communication may be detected by neighbour plants through belowground interactions and trigger its acclimation response (Fig 1). To test this, we designed an experiment in which maize leaves were briefly touched mimicking naturally occurring mechano-stimulation between neighbouring plants. A hydroponic system was used to disentangle the belowground interaction between maize siblings when above ground interactions between plants were prevented. This system was also chosen to avoid soil microbes that can alter and modify outcome of belowground interactions between related individuals [23]. By comparing root choice behaviour of the newly germinated seedlings, we evaluated whether the growth solution of touched plants can act as cues of neighbour identity and trigger plant response. The direct immersion of young maize plants in growth solution of previously removed touched plants aimed to test their acclimation response to sudden exposure. We next examined responses of plants that shared the same growth solution with touched neighbours, where aboveground interaction through volatiles was prevented.

## Materials and methods

### Plant material

As a model plant, we used *Zea mays* L. cultivar Delprim obtained from Delley Seeds and Plants Ltd. Delley, Switzerland. Before sowing, maize seeds were surface sterilized using a bleach solution (50% bleach: 50% distilled water) (commercial bleach 5%, Klorin) for five minutes, then rinsed thoroughly four times with distilled water. The sterilized seeds were germinated in Petri dishes between two layers of filter paper moisturized with distilled water. Petri dishes with seeds were placed in a growth chamber and then covered with black plastic pots to provide complete darkness.

Four days after the seed germination, 40 seedlings were selected that were as uniform in height as possible. One seedling was carefully placed into one of each four planting holes at the corners of a black cover. An additional small hole in the cover’s centre was used for the aeration tube connected to the water pump. The black cover was made of inert synthetic sponge material with minute pores, in order to allow the access of the nutrient solution around the seedlings. The cover with seedlings was then placed on the top of a plastic bucket (10 × 10 × 13 cm), previously autoclaved at 122°C for 20 min. The covers and air tubes were surface sterilized with 70% ethanol. Each bucket contained one litre of continuously aerated half-strength Hoagland solution (H2395-10L, Sigma-Aldrich); 0.08 g/L of Hoagland basal salts dissolved in distilled water. The solution provided the essential macro and micronutrients to the plants as described by Hoagland and Arnon [24]. The pH of the nutrient solution was 5.5. The temperature in the growing chamber was 20–22°C, with 60% relative humidity. Light cycle intervals were 16 hours of light supplied to plants from OSRAM white lamps (OSRAM FQ, 80 Watt, Ho constant lumix, Germany) with a light intensity of 220 μmol photons m^-2^ s^-1^, and 8 hours of darkness.

### Touch treatment

The touch treatment aimed to simulates the naturally occurring phenomenon: brief and light mechanical contact between leaves of neighbouring plants. Using method modified from Markovic et al. [21], all leaves of treated plants were gently touched from the base to the top. For this purpose, we used a soft squirrel hair face brush (Rouge) (Lindex, Sweden). Treated plants were touched one minute per day three hours after the beginning of the photoperiod. All leaves of treated plants remained undamaged at the end of the experiment as checked with Screening Electronic Microscope.

### Root choice test

**Fig 2:**
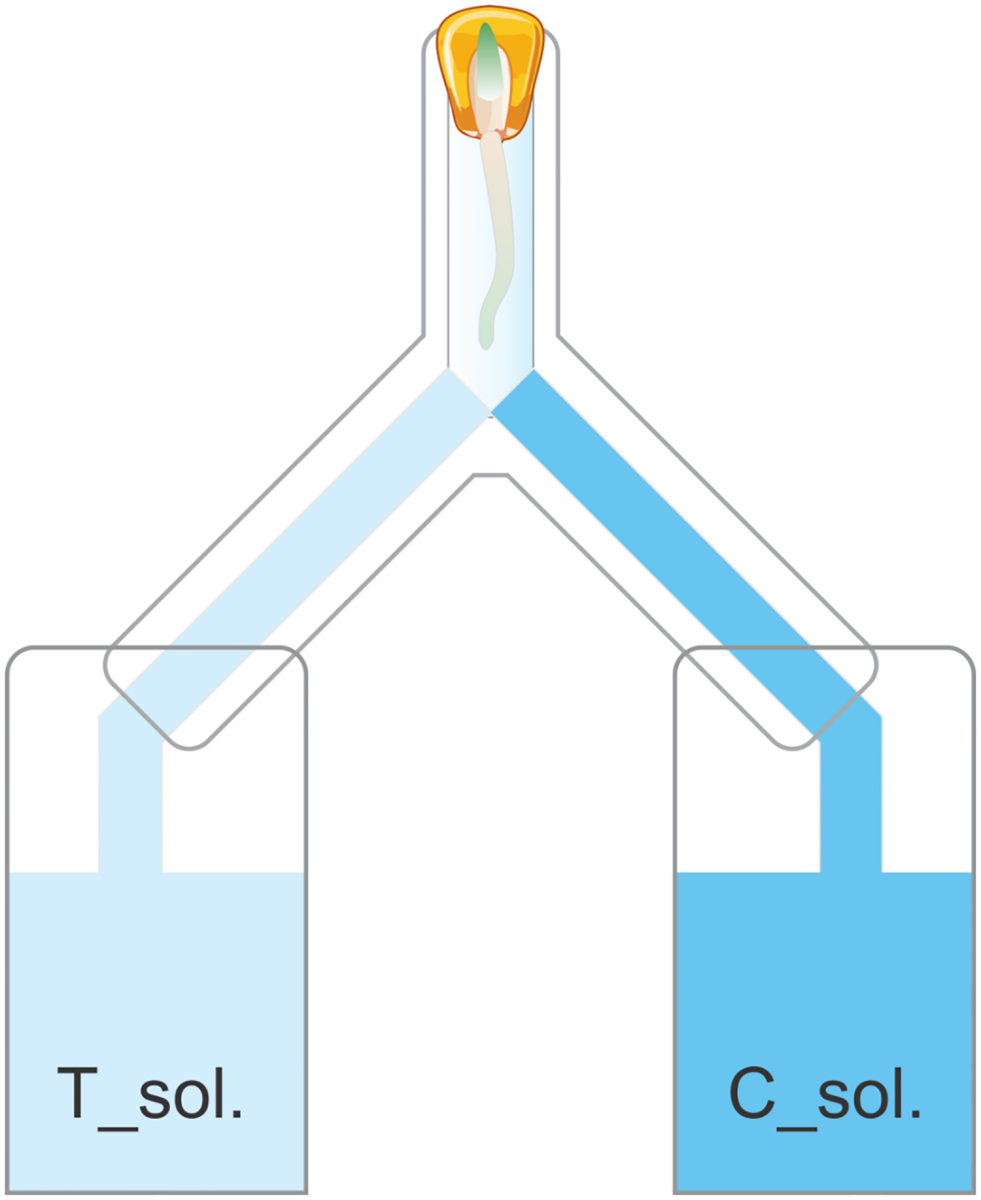
Root choice test in inverted Y tube where maize seedlings had a choice between growth solutions from previously touched plants (T_solution) and untouched controls (C_solution).

In this experiment, we tested the ability of the germinated seedling to choose between two spatial growth niches that contained the growth solution of either touched (T_solution) or untouched plants (C_solution) (Fig 2). Seeds of maize were germinated under the same above-mentioned conditions (see plant material). Two days after germination, each seed was placed inside the upper opening (1 cm, diameter) of the inverted Y-shape tube. All inverted Y-shape tubes were lined with filter paper and fixed into two 15ml conical centrifuge tubes from the bottom openings (Fig 2). The attached 15 ml tubes contained the growing solutions of the disposed plants obtained in the same way as for the transferring experiment (see below description of transferring experiment). One tube was filled with the treated growth solution (T_solution) and the other with the control growth solution (C_solution).

All parts were then fixed vertically on the outer wall of Perspex cages just for the purpose of support. Cages were covered with black plastic to provide darkness to the roots. All cages, afterwards, were placed in the same growing chamber as in all other conducted experiments. Three days after seeds were placed inside the invert Y-tubes, the direction (choice) of the main root of each seedling was recorded. Sixty-three seedlings were tested.

### Transferring experiment

**Fig 3:**
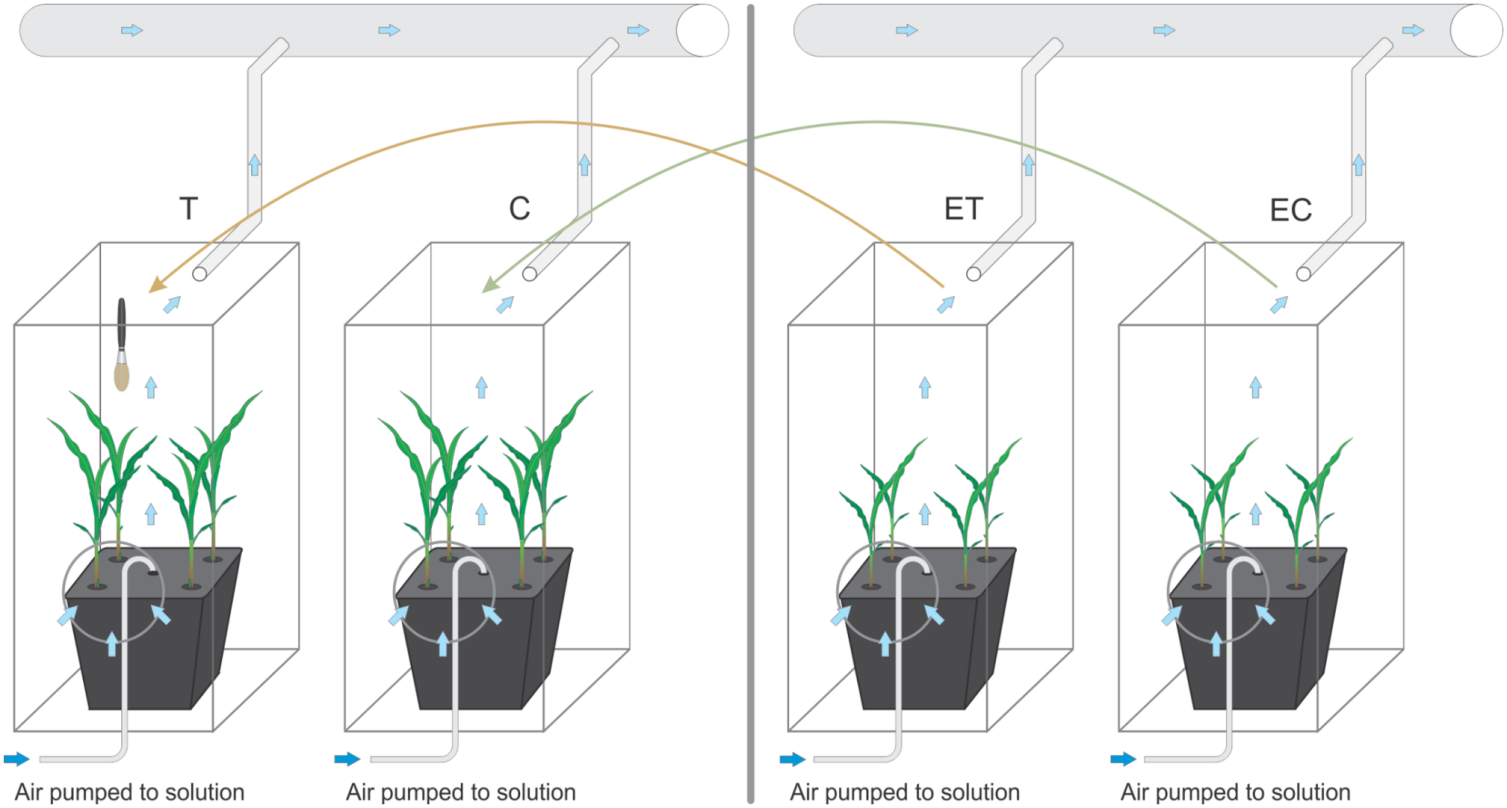
Graphical illustration of transferring experiment. Volatile interaction between treatments was prevented by covering each bucket with clean Perspex cages. T—touched plants, C—untouched plants, ET—plants transferred to growth solution of previously touched plants, EC—plants transferred to growth solution of untouched plants. Roots were constantly supplied with fresh air by an aquarium pump.

This experiment aimed to investigate the effect of sudden immersion of young maize seedlings into growth solution in which touched plants were previously grown. Five randomly distributed blocks were used in this experiment. Each block consisted of two buckets: one bucket with four treated plants (T_trans) and another bucket with four control plants (C_trans). To prevent volatile interaction between plants from the neighbouring buckets, each bucket was covered with a large modified clear Perspex cage (21 × 21 × 60 cm) with a front opening (15 cm in diameter). Air entered the system through the front opening, passed through the cage and sucked out through a Teflon tube attached to a vacuum tank at the top of the cage and then vented outside the growing chamber by a fan (Fig 3). An aquarium pump (Elite 801) with air output of 1000 mL min^-1^ was used to deliver oxygen into buckets. The touch treatment started 18 days after placing seedlings into a black cover (at the 4^th^ leaf stage) and lasted seven days. At the end of the touch treatment, all the plants in both treated and control buckets were carefully removed and replaced with nine days-old plants at the 2^nd^ leaf stage (ET_trans and EC_trans), which were grown under the same condition of the disposed ones in another growing chamber. The transferred plants were kept inside the cages without any extra treatments. After seven days, these plants from both treated (ET_trans) and control (EC_trans) buckets were harvested for biomass analysis.

### Sharing experiment

**Fig 4:**
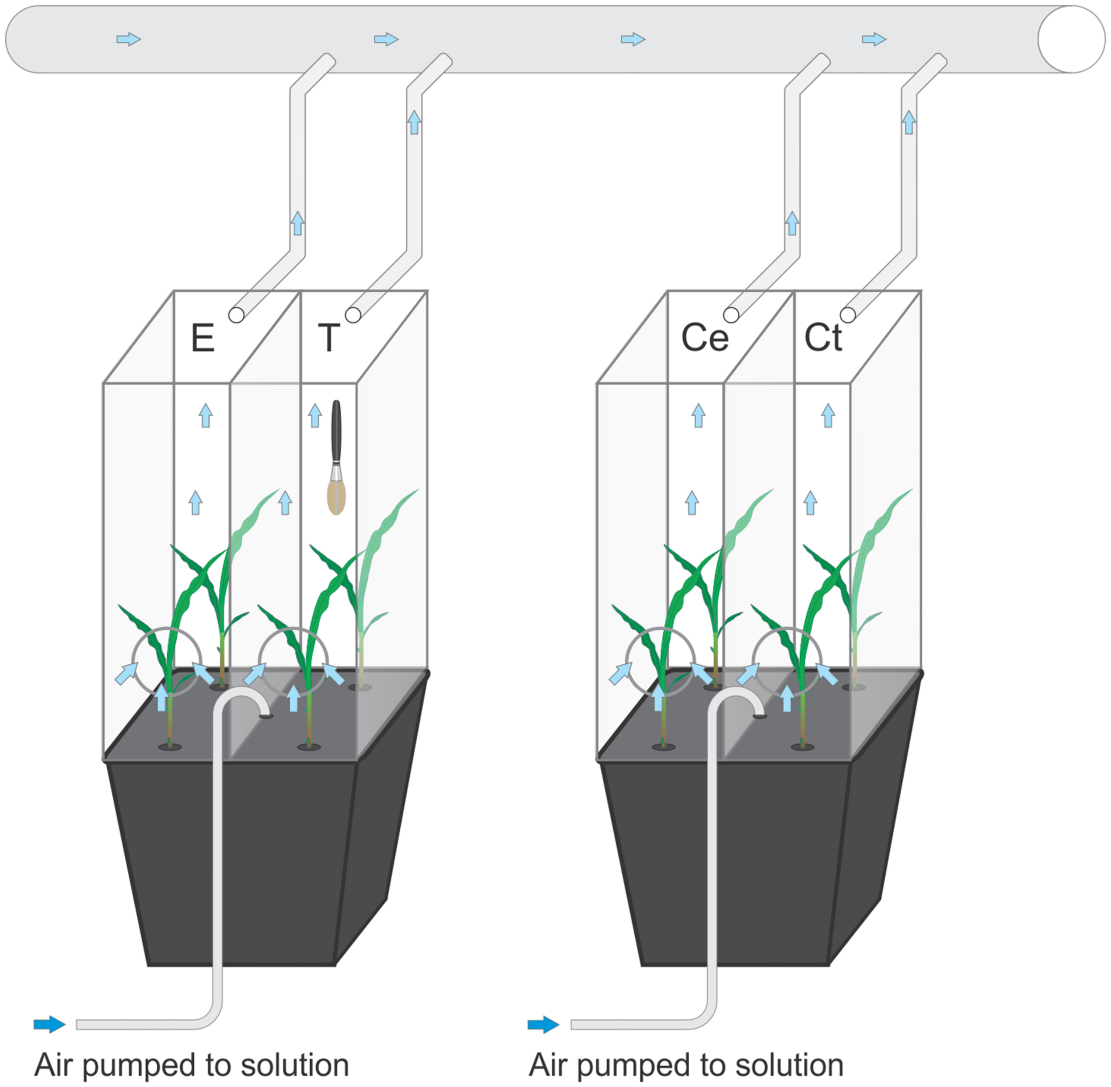
Graphical illustration of sharing experiment in which touched and control plants shared same solution. Volatile interaction between treatments was prevented by Perspex cages. T—touched plants, E—plants exposed to root exudates released by touched neighbors, Ce—control for E plants and Ct—control for T plants. Roots were constantly supplied with fresh air by an aquarium pump.

The experiment was conducted to test the plant’s ability to detect and acclimate to the changes in growth solution induced by touching between nearby neighbours. Both touched and exposed plants shared the same growth solution from the beginning of the experiment. Eight randomly distributed blocks with a total of 64 plants were used. Each block had treated bucket comprised of two different treatments, two “touched” plants (T_share) and two “exposed” neighbours (E_share) and one bucket with four control plants (C_share) (Fig 4). All treatments were randomly distributed within the blocks. Two clear Perspex cages (10 × 10 × 40 cm) were placed on the top of each bucket. One cage was placed over the touched plants (T_share), and the other over the exposed neighbours (E_share) plants to prevent any potential volatile interaction between the two pairs. The same procedures were carried out in the control (C_share) buckets (Fig 4). Touch treatment started 5 days after placing buckets in the growing chamber (at the 2^nd^ leaf stage). After six days of treatment, all plants were harvested for biomass analysis.

### Analysed morphological parameters

At the end of transferring and sharing experiments, plants were cut above the ground and separated into stems, leaves and roots. The roots of each plant were carefully washed and cleaned with water. Stems, leaves and roots of each plant were scanned separately utilizing a dual lens scanner (Epson 4490Pro). Leaf surface area (LA), and stem height (SH) were measured using WinRHIZO image analysis system. By employing the same program, roots were divided into seven classes according to diameter (0 < D < 0.25; 0.25 ≤ D < 0.42, 0.42 ≤ D < 0.60; 0.60 ≤ D < 1.0; 1.0 ≤ D < 1.5; 1.5 ≤ D < 2.0; ≥ 2.0) [25]. Root parameters (length, average diameter and volume) for each root class were measured. Leaves, stems, and roots from each plant were separately packed into labelled aluminium bags. After drying for 48 h at 70°C, samples were kept for 24 h at room temperature before they were weighed. These data i.e. total dry weight (TDW), stem dry weight (SDW) and leaf dry weight (LDW) were used to calculate plant biomass fractions, i.e. leaf mass fraction (LMF), stem mass fraction (SMF) and root mass fraction (RMF). In addition, some growth indices i.e. specific leaf area (SLA) and the shoot-root ratio (S/R ratio), were also calculated.

### UHPLC-MS analyses of root exudates

Root exudates were centrifuged in 1.5-mL plastic tubes and the supernatants transferred to vials for analysis by ultrahigh-performance liquid chromatography—mass spectrometry (UHPLC-MS). Additionally, root exudates (30.0 mL) were loaded onto 1-g C-18 SPE columns and the columns were washed with 5 mL water. Subsequently, each column was eluted with 6 mL MeOH and the extracts were dried in glass tubes in a vacuum centrifuge, redissolved in 300 uL MeOH and transferred to vials for UHPLC-MS analysis.

UHPLC-MS was carried out on an Agilent 1290 Infinity II system (Agilent, Palo Alto, CA, USA) connected to a maXis Impact quadrupole time-of-flight mass spectrometer (QTOF-MS) (Bruker Daltonic GmbH, Bremen, Germany) via an electrospray ionization interface. Analyses were performed both in positive and negative mode with a scanning of m/z 50–1500, and mass spectra were calibrated against sodium formate clusters injected at the beginning of each analysis. The separation was achieved on an Accucore Vanquish column (C-18, 1.5 μm, 2.1 × 50 mm, Thermo Scientific, Waltham, MA, USA) at a flow rate of 0.9 mL min^-1^ and 2 μL was injected to each sample. The mobile phases were water (A) and acetonitrile (B), both with 0.2% formic acid, and the linear gradient was: 5–95% B in 3 min followed by 95% B for 1.2 min. Blank MeOH samples were injected before and after analysis of the samples.

The software Compass DataAnalysis 4.3 (Bruker Daltonic) was used to calibrate the MS raw data against the sodium formate clusters and to convert the data to mzXML format. Ion-chromatogram peak picking was done in the software environment R by the program XCMS using the centWave method [26–28] and the resulting peak-areas of the molecular features were normalized against sample biomass (fresh and dry root biomass, as well as fresh and dry full plant biomass, were used).

### Statistical analyses

To test whether a root chose one of the two alternatives presented in a test significantly more often than expected by chance, the binomial test with 0.5 expected probabilities was used which tests for differences in random choice indicating preference [29].

For statistical analyses of morphological parameters were used mixed statistical models [30]. The models for data analyses of transferring experiment included treatments (T_trans and C_trans) as fixed effects, and block and tank*block as random effects. For the sharing experiment, the models included treatments (E_share, T_share and C_share) as fixed effects, and block, block*treatment, block*tank and block*tank*chamber as random effects. Similar, but not identical, results (not reported here) were obtained in an analysis of the mean values within each column. The assumptions underlying the analysis were checked by preparing diagnostic plots. No apparent deviations from the assumptions were detected. The Mixed procedure of the SAS package was used for the analyses [31]. Least squares means were calculated and compared using Tukey’s HSD test.

Statistical analysis of root exudates was performed using MetaboAnalyst 3.5, a web-based tool suite for metabolomic data analysis [32]. Following Pareto scaling (division by the square root of the standard deviation of the respective variable), differences between treated plants and control plants were analyzed by partial least squares—discriminant analysis (PLS-DA), Welch’s t-test and by the construction of heat-maps.

## Results

### Root choice test

**Fig 5:**
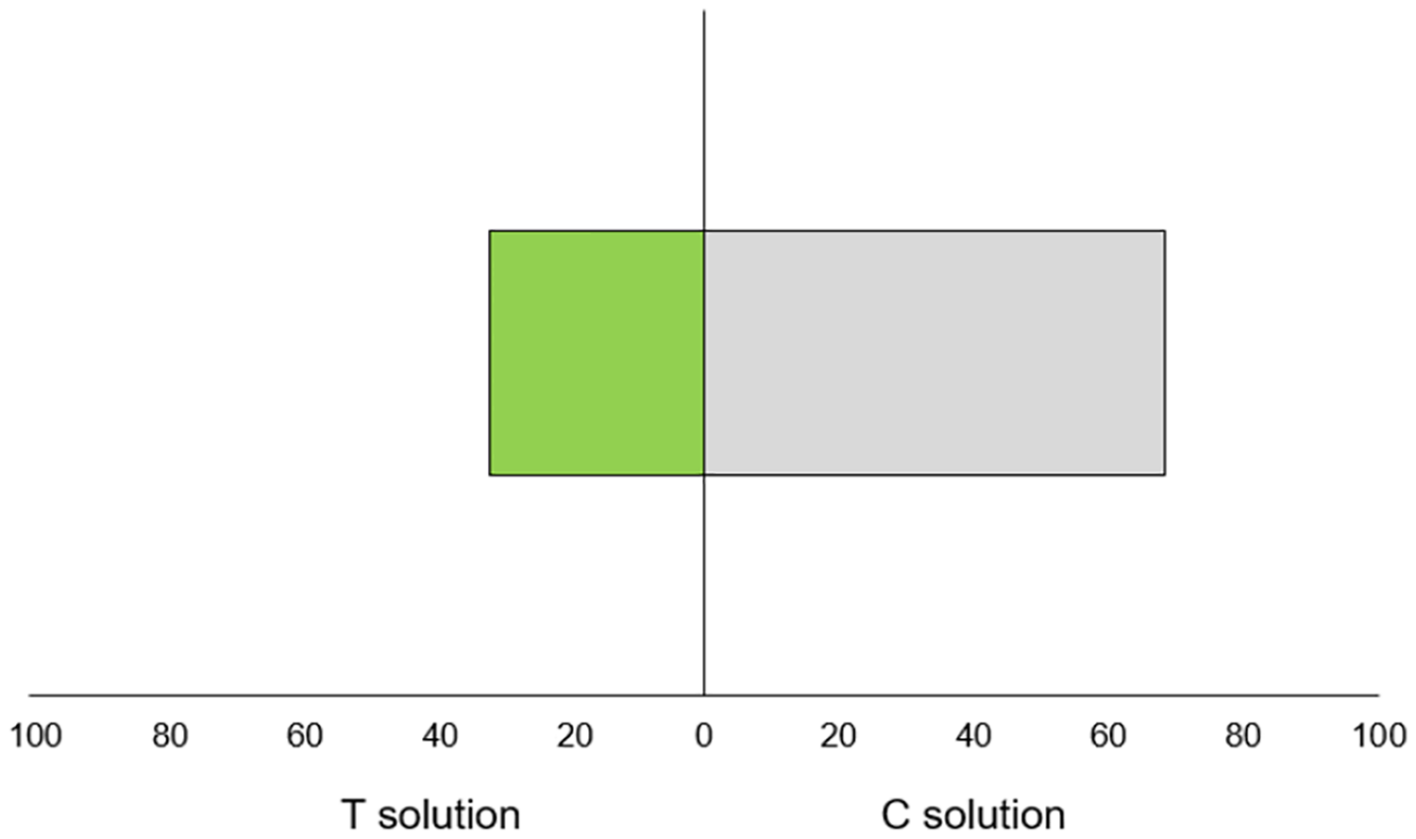
The choice frequency of newly emerged main maize root between the growth solution of touched (T solutions) and un-touched plants (C solution) (P = 0.005, binomial test with 0.5 expected probabilities).

The primary root of newly emerged maize seedlings grew significantly more often towards the growth solution from control plants than towards the solution from the stressed plants (P = 0.005) (Fig 5). This result demonstrates root capacity to actively distinguish between different growth solutions. There were also occasions when roots went toward growth solutions of touched plants but later changed direction towards the growth solution from control plants, but the opposite, roots changing direction from control to touched plants solutions, was not observed.

### Transferring experiment

**Fig 6:**
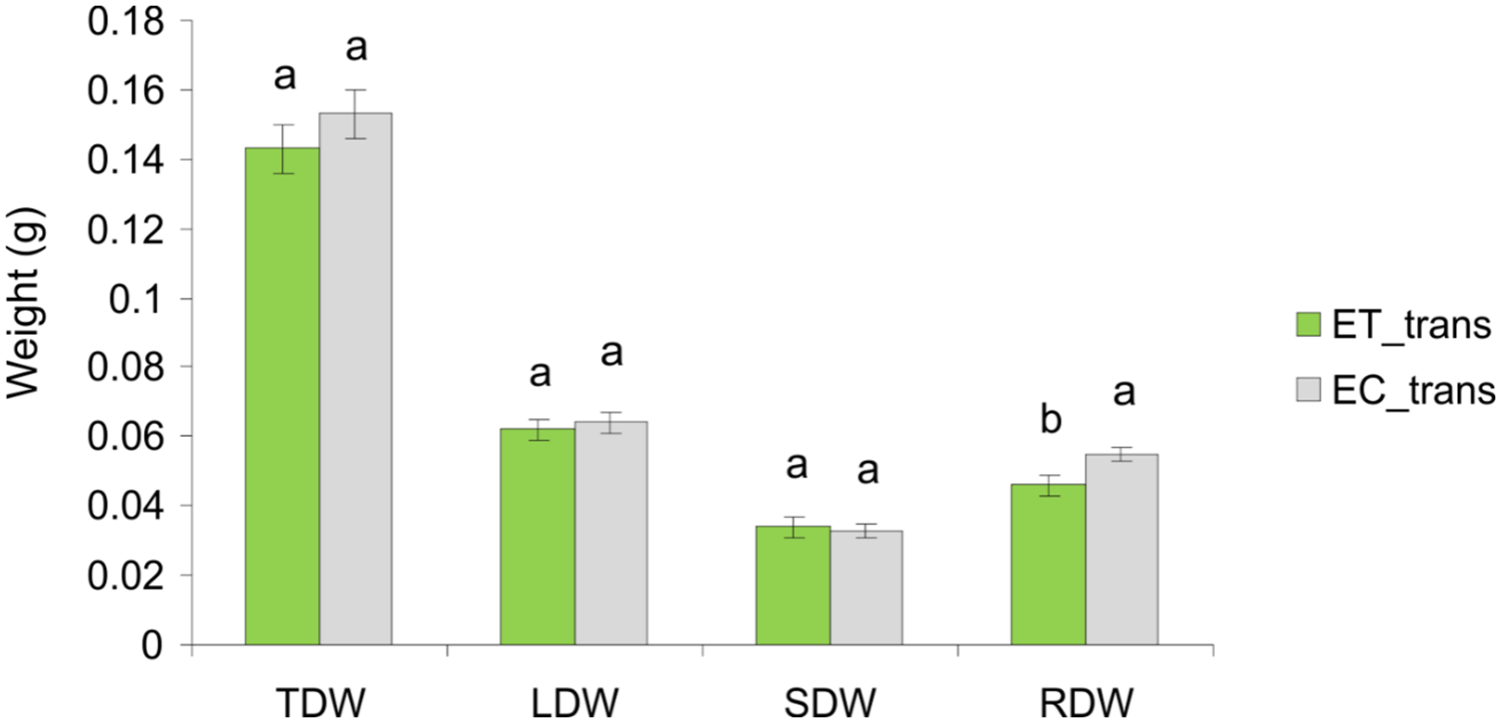
Changes in the biomass production and biomass allocation patterns between plants exposed to the treated solution (ET_trans) and plants exposed to the control solution (EC_trans). Different letters above each variable represent significant difference between treatments.

**Fig 7:**
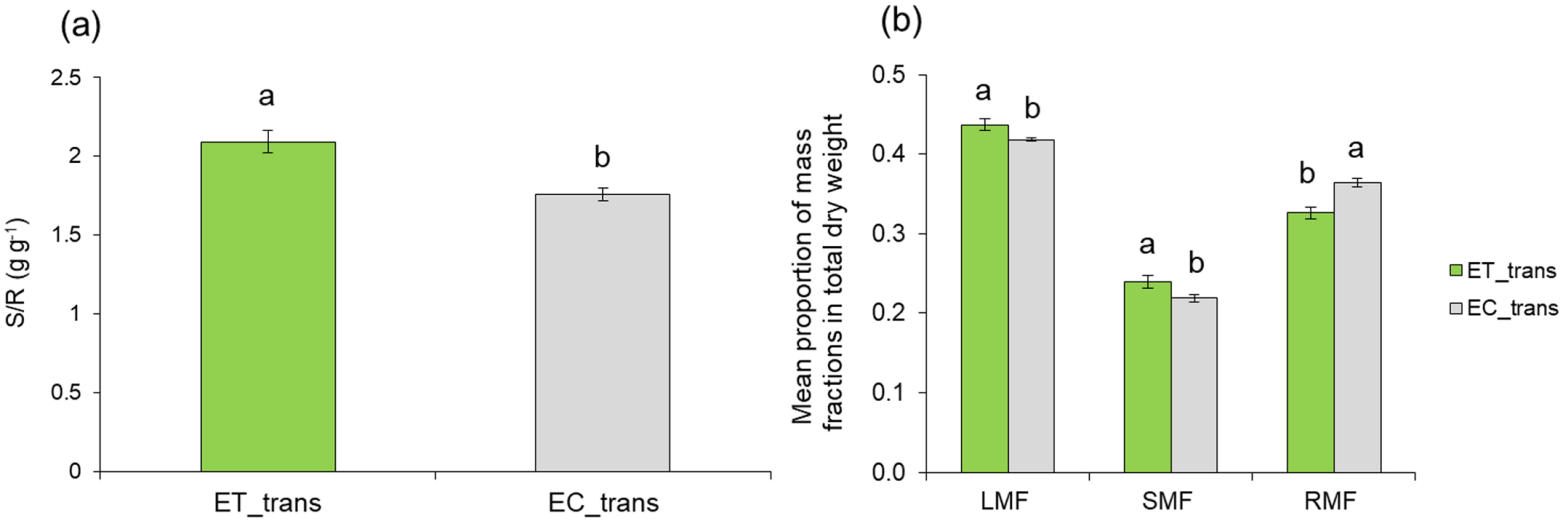
Changes in a) S/R ratio and b) biomass allocation patterns between plants transferred to the growth solution of touched (ET_trans) and control plants (EC_trans). Different letters above each variable represent significant difference between treatments, while ns means no significant difference between treatments.

Young maize seedlings (ET_trans) directly transferred to growth solution of touched plants T_solution had significantly changed the pattern of biomass allocation compared to controls (EC_trans) exposed/transferred to C_solution. Although no changes in TDW was observed between the treatments (F_1,30_ = 1.28, *P = 0*.*27*), RDW of ET_trans plants was lower than EC_trans plants (F_1,30_ = 8.08, *P* = 0.008) (Fig 6). Subsequently, the ET_trans plants had higher S/R ratio (F_1,30_ = 7.13, *P = 0*.*0121*), allocating more biomass to the aboveground organs (Fig 7a), which resulted in higher LMF (F_1,30_ = 5.93, *P* = 0.018), SMF (F_1,30_ = 4.16, *P* = 0.05) and lower RMF (F_1,30_ = 7.62, *P* = 0.001) compared to EC_trans (Fig 7b).

Changes in root parameters/fractions between the treatments were also observed. In general, ET_trans plants had less root volume (F_1,30_ = 5.37, *P* = 0.03) than EC_trans plants. In particular, ET_trans had shorter axial roots (D ≥ 2 mm) than EC_trans (F_1,30_ = 9.02, *P* = 0.005) (S1 Table).

### Sharing experiment

**Fig 8:**
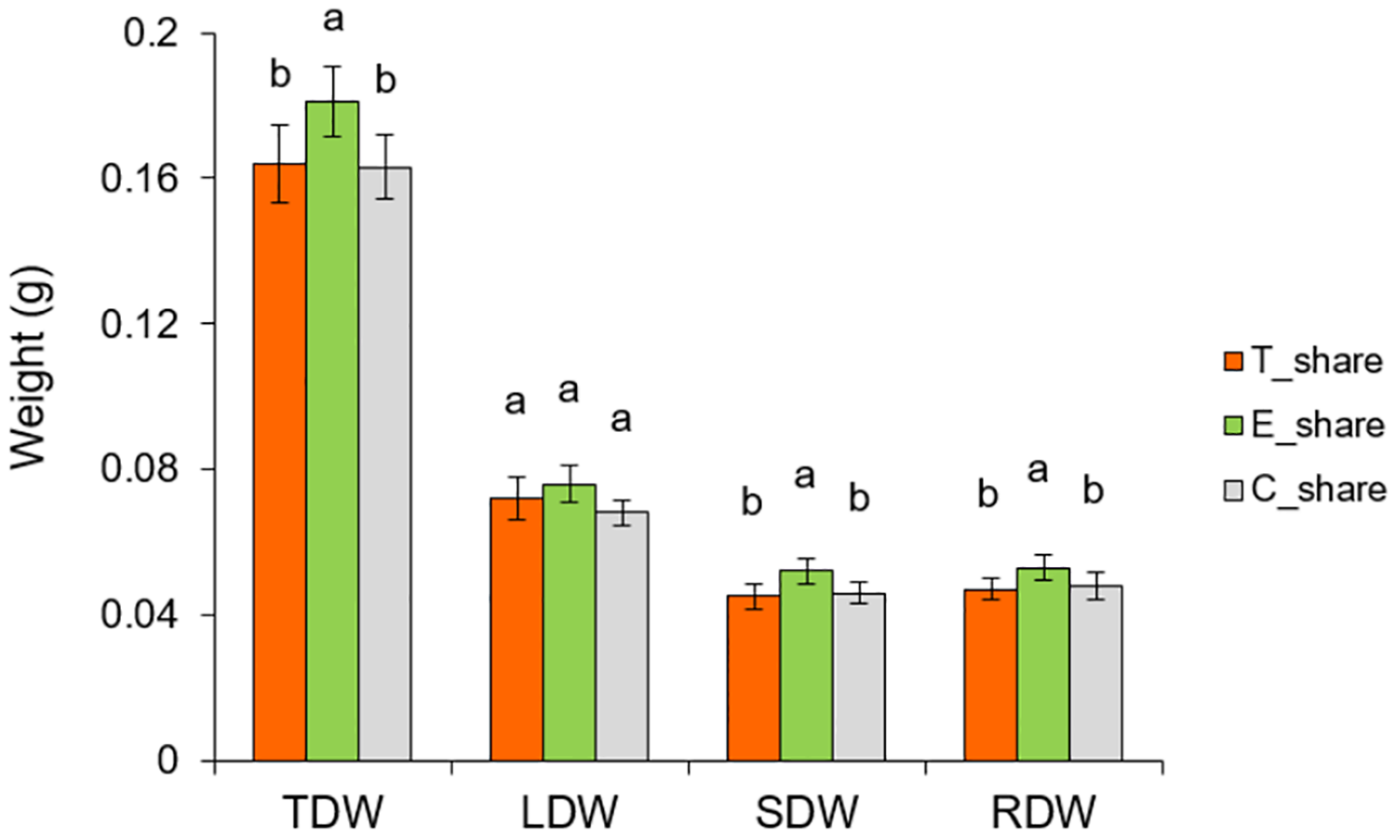
Changes in the biomass production and biomass allocation patterns between touched plants (T_share) and those exposed to touched (E_share) and untouched neighbours (C_share). Different letters above each variable represent significant difference between treatments, while ns means no significant difference between treatments.

**Fig 9:**
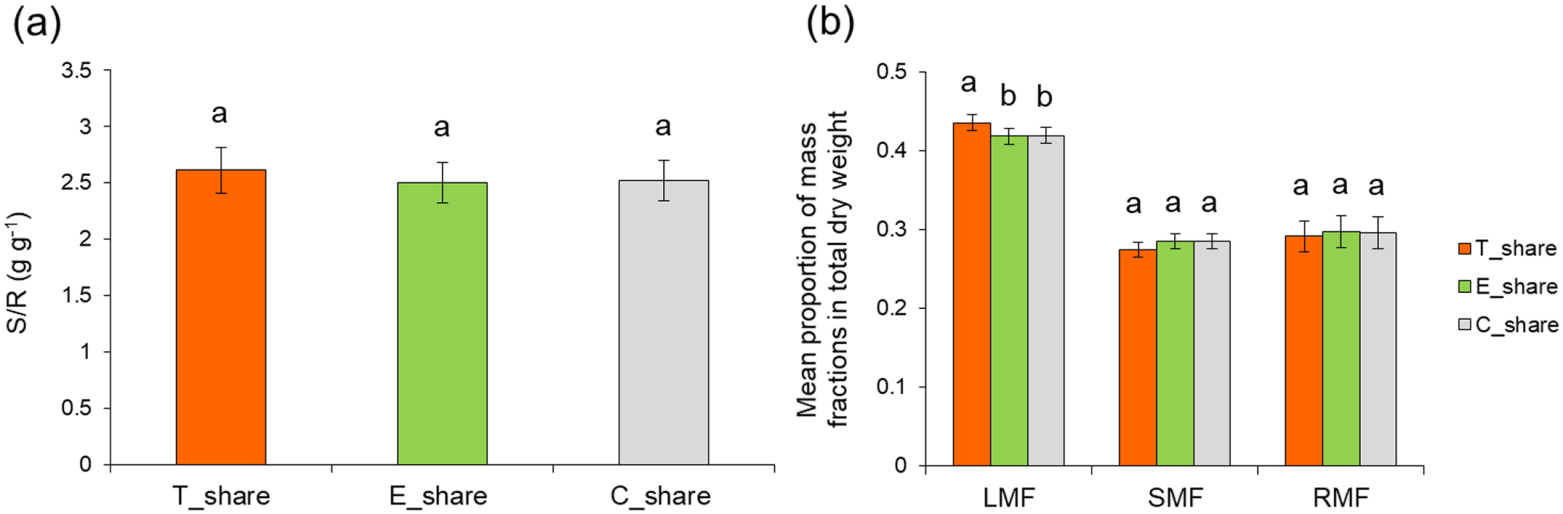
Changes in a) S/R ratio and b) biomass allocation patterns between touched plants (T_share), exposed to touched (E_share) and control plants (C_share). Different letters above each variable represent significant difference between treatments, while ns means no significant difference between treatments.

Non-touched plants (E_share) that shared the growth solution with touched plants (T_share), produced significantly higher TDW compared to touched (T_share) (*P* = 0.02) and control plants (C_share) grown in the separate buckets (*P* = 0.03) (Fig 8). Receiving non-touched plants invested significantly more resources to SDW compared to touched and non-touched control plants (*P* = 0.006; *P* = 0.02) respectively, and more to RDW in contrast to touched plants (*P* = 0.02) but not to control plants (*P* = 0.06). The pattern of biomass distribution did not affect S/R ratio comparing all three treatments, indicating that root exudates stimulate growth (Fig 9a). However, the LMF of touched plants was significantly increased compared to non-touched sharing plants (*P* = 0.03) and to control plants (*P* = 0.03) (Fig 9b).

Root parameters/fractions did not change between these treatments except for the root length of the 3^rd^ diameter class (0.42 ≤ D < 0.60 mm), which is related to the lateral roots. In this diameter class, roots of touched plants (T_share) were significantly shorter than those of control plants (C_share) (*P* = 0.02), but not from those of exposed plants (E_share) (*P* = 0.26) (S2 Table).

### Root exudates

No significant differences in metabolite composition of the root exudates from treated and control plants were detected, regardless if positive or negative MS analysis was performed, or if concentrated (100-fold) or non-concentrated samples were analyzed, or how sample normalization was done. This can be due to many reasons, including; i) too low concentration of the root exudate components responsible for the observed differences on the plant level, ii) loss of possibly volatile or unstable active components during sample pretreatment, or iii) the active components falling outside the scanned m/z window (50–1500) during UHPLC-MS analysis.

## Discussion

Our results show that the above ground plant-plant communication by brief touch can provoke responses in nearby non-touched plants through belowground communication. This indicates that responses to neighbouring plants can be significantly affected by the physical conditions (in this case, mechano-stimulation) to which these neighbours are exposed to. It thus suggests that plant-plant belowground communication is modified by above-ground mechanical stimulation.

Touch is one of the most common mechanical stimuli in higher plants and is known to induce strong morphogenetic changes over time. A recent study has demonstrated that brief touching among neighbouring plants can be used as a cue in the detection of potential competitors [22]. As plants grow in communities closely associated with other plants, they constantly monitor specific cues that may occur above- or belowground. There is increasing evidence that these cues not only indicate neighbour presence but also provide information about the identity of the neighbours [33]. With the respect to belowground cues, roots demonstrated to possess complex patterns of growth behaviour [34]. Our study clearly shows that roots of very young maize seedlings pose an extraordinary capacity to quickly detect changes in cues vectored by growth solution directing roots away from neighbours exposed to brief mechano stimuli (Fig 5). In this way, roots may detect the changed physiological status of neighbours through the perception of cues they release, even if chemical analyzes did not show significant changes in metabolite composition. Observed early plastic responses of roots demonstrate that plants actively participate in social interactions with nearby neighbours in which belowground cues from touch neighbours play an important role. Although resources analysis was missing in our experiments, there are strong reasons to assume our results are not mediated through resource availability. If the observed morphological changes were resource-based, we would expect that the newly germinated seedlings in the choice test to grow more toward the solution from touched plants, as touched (stressed) plants grew less, thus have fewer resources uptake [35] leaving more nutrient in their growth solution. In addition, for the same reason we expected from ET_trans plants to produce more biomass than EC_trans, but these changes were not observed.

The ability of plants to rapidly detect and respond to changes in their surrounding environment is essential as it determines their survival [36]. Depending on the type of exposure, maize plants expressed high levels of plasticity in response to the belowground cues from touched plants. Direct transplantation of young seedlings into growth solution of touched plants triggered an increase in S/R ratio while TDW was not different (Figs 6 and 7a). This raises the question why would plants produce relatively fewer roots or grow roots away from neighbour plants that are touched. One explanation could be that touching is a cue for impending competition, as plants growing closer are more likely to touch each other. It has been demonstrated in *Arabidopsis thaliana* that plants exhibit neighbour induced shade avoidance (e.g. petiole elongation and leaf hyponasty) only after touching neighbours [19]. In this study, it was the target plant itself being touched while the target plant responds to the fact that its neighbour is touched [19]. All the same, even neighbours being touched could be a sign of impending competition. However, touch could also be due to other causes (e.g. herbivores) and thus signal other forms of stress.

Under mechanical stresses, plants adjust their morphological [37] and physiological characteristics [38] as they tend to allocate more biomass to the stressed tissues [37, 39]. Such response was also observed in our study on touched plants (T_share) that resulted in an increase of LMF (Fig 9b). This type of allocation indicates the existence of a specific pattern of biomass distribution between organs of the same plant that aim to acclimate to the given situation. Plant response to mechano stimuli can also alter the synthesis of chemical cues [10] that may have informative value for the surrounding neighbours. In such situation, eavesdropping plants can exploit neighbour cues to detect them and adequately prepare for competitive scenarios [7, 40]. We showed that plants can respond not only to the presence and identity of the neighbours but also to their physiological status/stress condition. The ability of plants to modify their growth and morphology is fundamental to reproductive performance and fitness [41]. The fact that exposed plants (E_share) perceive and respond to changes in the growth solution from genetically identical neighbours suggest that the mechanism involves touch-induced root exudates as a signalling vector that conveys specific information about the emitter. Therefore, in such situation, touch provides an extra indication of the neighbour presence and their physiological status. A previous study has shown that physiological changes in infested plants by pea aphid *Acyrthosiphon pisum* can influence its non-infested neighbours through root-root interaction to be more attractive to parasitoid *Aphidius ervi* [42]. As the touched and exposed plants shared the same growth solution, detection and response observed in receivers are not regulated by resource availability. This can also indicate the existence of highly sophisticated perception system in maize roots which enable them to discriminate between belowground cues and respond differentially in accordance with actual stress status of genetically identical neighbours. This sort of highly sophisticated responses to touch-induced changes in growth solution suggest the existence of remarkable plasticity in terms of biomass distribution by which plants alter their morphology based on the perception of particular cues that reveals the stress status of its closest neighbours. An earlier study showed that unstressed *Pisum sativum* plants are able to perceive and respond to stress cues emitted by roots of stressed neighbours and in turn induce stress responses in further unstressed plants [43]. Therefore, it is quite reasonable to hypothesize that cues from root exudates of touched plants could also be exchanged among neighboring plants and therefore influence plant interactions at even longer distances.

Traits expressed in direct exposure to growth solution in which previously touched plants were grown indicate stress avoidance response, while traits expressed in the shared growth solution indicate acclimation to given situation. Two different responses in plants growth demonstrate that the changes in the belowground environment can provide cues able to elicit distinct patterns of developmental plasticity depending on the type of exposure. Our results suggest the existence of another mechanism in plant-plant communication by which mechano stimuli perceived by the leaves affect belowground plant interactions. This adds a new dimension to the functional role of touch to induce cues that can modify belowground interaction with proximate neighbours.

## Conclusions

In conclusion, this study reveals a new level of complexity in below-ground plant-plant interactions showing that the direction and extent of plant root responses to neighbours can be affected by the above-ground physical stress to which neighbours are exposed. In addition to highlighting the complexity of plant-plant interactions, these results also entail that interpretation of results in experiments on plant-plant interactions should take into account the extent to which plants are touched during the experiment, as they often are when one conducts measurements on them. However, the ecological significance of the observed responses still needs to be further explored.

## Supporting information

S1 Table Root fractions of ET_trans and EC_trans plants. (DOCX)

S2 Table Root fractions of T_share, E_share and C_share plants. (DOCX)

S1 File Data root choice test. (XLSX)

S2 File Transfer experiment data. (XLSX)

S3 File Sharing experiment data. (XLSX)

S4 File Data of chemical analyses of root exudates experiment data. (XLSX)

S5 File Data of chemical analyses of root exudates experiment data. (XLSX)
