# Supplementary material for: Aboveground mechanical stimuli affect belowground plant-plant communication
Source: PLoS One. 2018 May 2;13(5):e0195646. doi: 10.1371/journal.pone.0195646 (PMC5931455; doi:10.1371/journal.pone.0195646)
Supplement: S1 Table — (DOCX) [file pone.0195646.s001.docx]

**Table S1**. Changes in root fractions of ET_trans and EC_trans plant divided into seven classes according to diameter (0 < D < 0.25; 0.25 ≤ D < 0.42, 0.42 ≤ D < 0.60; 0.60 ≤ D < 1.0; 1.0 ≤ D < 1.5; 1.5 ≤ D < 2.0; ≥ 2.0). Significant levels: *P ≤ 0.05, **P <0.01, ***P <0.001, and ns P > 0.5.

|  | **ET_trans** | **EC_trans** | ***t* value** ^significance levels^ |
| --- | --- | --- | --- |
|  | (Mean ± SE) | (Mean ± SE) |  |
| **Avg. Diameter (mm)** | 0.524 ± 0.01 | 0.498 ± 0.02 | -0.63 ^ns^ |
| **Length/volume (cm/m3)** | 314.0 ± 13.4 | 466.9 ± 57.8 | 1.25 ^ns^ |
| **Root Volume (cm3)** | **0.681 ± 0.03** | **0.796 ± 0.03** | **2.32^**^** |
| **Root Surface area (cm^2^)** | 18.79 ± 0.87 | 24.28 ± 1.70 | 1.5 ^ns^ |
| **Total root length (cm)** | 313.7 ± 13.4 | 466.5 ± 57.8 | 1.25 ^ns^ |
| **length 1^st^ class (cm)** | 102.5 ± 5.35 | 180.8 ± 39.0 | 0.95 ^ns^ |
| **(0 < D < 0.25 mm)** |  |  |  |
| **Length 2^nd^ class (cm)** | 97.19 ± 5.04 | 151.6 ± 16.6 | 1.6 ^ns^ |
| **(0.25 ≤ D < 0.42 mm)** |  |  |  |
| **Length 3^rd^ class (cm)** | 18.54 ± 1.60 | 28.48 ± 2.96 | 1.94 ^ns^ |
| **(0.42 ≤ D < 0.60 mm)** |  |  |  |
| **Length 4^th^ class (cm)** | 50.15 ± 3.19 | 57.79 ± 2.76 | 1.19 ^ns^ |
| **(0.60 ≤ D < 1.0 mm)** |  |  |  |
| **Length 5^th^ class (cm)** | 39.66 ± 2.33 | 40.56 ± 1.86 | 0.2 ^ns^ |
| **(1.0 ≤ D < 1.5 mm)** |  |  |  |
| **Length 6^th^ class (cm)** | 4.084 ± 0.46 | 5.151 ± 0.53 | 1.74^ns^ |
| **(1.5 ≤ D < 2.0 mm)** |  |  |  |
| **Length 7^th^ class (cm)** | **1.589 ± 0.13** | **2.075 ± 0.15** | **3^***^** |
| **(D ≥ 2.0 mm)** |  |  |  |
