# Supplementary material for: Aboveground mechanical stimuli affect belowground plant-plant communication
Source: PLoS One. 2018 May 2;13(5):e0195646. doi: 10.1371/journal.pone.0195646 (PMC5931455; doi:10.1371/journal.pone.0195646)
Supplement: S2 Table — (DOCX) [file pone.0195646.s002.docx]

**Table S2**. Changes in root fractions of T_share, E_share and C_share plant were divided into seven classes according to diameter (0 < D < 0.25; 0.25 ≤ D < 0.42, 0.42 ≤ D < 0.60; 0.60 ≤ D < 1.0; 1.0 ≤ D < 1.5; 1.5 ≤ D < 2.0; ≥ 2.0). Significant levels: *P ≤ 0.05, **P <0.01, and ns P > 0.5.

|  | **T_share** | **E_share** | **C_share** | ***t* value**  **^significance levels^** | | |
| --- | --- | --- | --- | --- | --- | --- |
|  | (Mean ± SE) | (Mean ± SE) | (Mean ± SE) | C-E | C-T | E-T |
| **Avg. Diameter (mm)** | 0.397 ± 0.01 | 0.416 ± 0.02 | 0.395 ± 0.008 | -1.05 ^ns^ | -0.11 ^ns^ | 1.16 ^ns^ |
| **Length/volume (cm/m^3^)** | 709.0 ± 64.10 | 729.5 ± 64.99 | 753.0 ± 53.20 | 0.32 ^ns^ | 0.6 ^ns^ | 0.4 ^ns^ |
| **Root Volume (cm^3^)** | 0.846 ± 0.05 | 0.912 ± 0.05 | 0.897 ± 0.05 | -0.36 ^ns^ | 1.49 ^ns^ | 1.79 ^ns^ |
| **Root Surface area (cm^2^)** | 28.03 ± 1.87 | 29.89 ± 1.91 | 29.49 ± 1.6 | -0.21 ^ns^ | 0.75 ^ns^ | 1.25 ^ns^ |
| **Total root length (cm)** | 708.3 ± 64.04 | 728.7 ± 64.90 | 752.3 ± 53.10 | 0.32 ^ns^ | 0.6 ^ns^ | 0.4 ^ns^ |
| **Length 1^st^ class (cm)** | 308.4 ± 35.39 | 286.9 ± 31.65 | 297.1 ±26.97 | 0.23 ^ns^ | -0.25 ^ns^ | -0.68 ^ns^ |
| **(0 < D < 0.25 mm)** |  |  |  |  |  |  |
| **Length 2^nd^ class (cm)** | 247.5 ± 23.71 | 276.8 ± 28.97 | 286.1 ± 20.82 | 0.32 ^ns^ | 1.32 ^ns^ | 1.36 ^ns^ |
| **(0.25 ≤ D < 0.42 mm)** |  |  |  |  |  |  |
| **Length 3^rd^ class (cm)** | **56.48 ± 10.77** | **69.43 ± 10.77** | **80.06 ± 9.44** | **1.18 ^ns^** | **2.63^**^** | **1.25 ^ns^** |
| **(0.42 ≤ D < 0.60 mm)** |  |  |  |  |  |  |
| **Length 4^th^ class (cm)** | 50.66 ± 5.86 | 46.62 ± 3.72 | 41.02 ± 2.52 | -1.19 ^ns^ | -2.04 ^ns^ | -0.74 ^ns^ |
| **(0.60 ≤ D < 1.0 mm)** |  |  |  |  |  |  |
| **Length 5^th^ class (cm)** | 36.25 ± 2.59 | 39.37 ± 1.75 | 38.58 ± 2.85 | -0.26 ^ns^ | 0.76 ^ns^ | 0.89 ^ns^ |
| **(1.0 ≤ D < 1.5 mm)** |  |  |  |  |  |  |
| **Length 6^th^ class (cm)** | 6.678 ± 0.82 | 7.650 ± 0.71 | 7.203 ± 0.52 | -0.61 ^ns^ | 0.71 ^ns^ | 1.14 ^ns^ |
| **(1.5 ≤ D < 2.0 mm)** |  |  |  |  |  |  |
| **Length 7^th^ class (cm)** | 2.263 ± 0.26 | 1.905 ± 0.08 | 2.182 ± 0.17 | 1.16 ^ns^ | -0.34 ^ns^ | -1.3 ^ns^ |
| **(D ≥ 2.0 mm)** |  |  |  |  |  |  |
